# New Insight on Epidemiology and Management of Bacterial Bloodstream Infection in Patients with Hematological Malignancies

**DOI:** 10.4084/MJHID.2015.044

**Published:** 2015-07-01

**Authors:** Sara Lo Menzo, Giulia la Martire, Giancarlo Ceccarelli, Mario Venditti

**Affiliations:** Department of Public Health and Infectious Diseases. University of Rome “Sapienza”, Rome (Italy)

## Abstract

Bloodstream infections (BSI) are a significant cause of morbidity and mortality in onco-hematologic patients. The Gram-negative bacteria were the main responsible for the febrile neutropenia in the sixties; their impact declined due to the use of fluoroquinolone prophylaxis. This situation was followed by the gradual emergence of Gram-positive bacteria also following the increased use of intravascular devices and the introduction of new chemotherapeutic strategies. In the last decade, the Gram-negative etiology is raising again because of the emergence of resistant strains that make questionable the usefulness of current strategies for prophylaxis and empirical treatment. Gram-negative BSI attributable mortality is relevant, and the appropriate empirical treatment significantly improves the prognosis; on the other hand the adequate delayed treatment of Gram-positive BSI does not seem to have a high impact on survival. The clinician has to be aware of the epidemiology of his institution and colonizations of his patients to choose the most appropriate empiric therapy. In a setting of high endemicity of multidrug-resistant infections also the choice of targeted therapy can be a challenge, often requiring strategies based on off-label prescriptions and low grade evidence.

In this review, we summarize the current evidence for the best targeted therapies for difficult to treat bacteria BSIs and future perspectives in this topic. We also provide a flow chart for a rational approach to the empirical treatment of febrile neutropenia in a multidrug resistant, high prevalence setting.

## Emerging Bacterial Infection in Hematological Neutropenic Patients

Although in the last decades noteworthy improvements have been achieved in the management of hematologic cancer patients, infections persist as leading cause of morbidity and mortality particularly during the cytotoxic neutropenia, defined as a neutrophil count < 500/mmc.^1^,^2^ Respiratory tract infections occur very often, followed by bloodstream infections (BSI), urinary tract infections, skin/skin structure infections and oro-pharynx/gastrointestinal tract infections.^2^ In this paper, we shall focus only on BSI.

**Figure 1:**
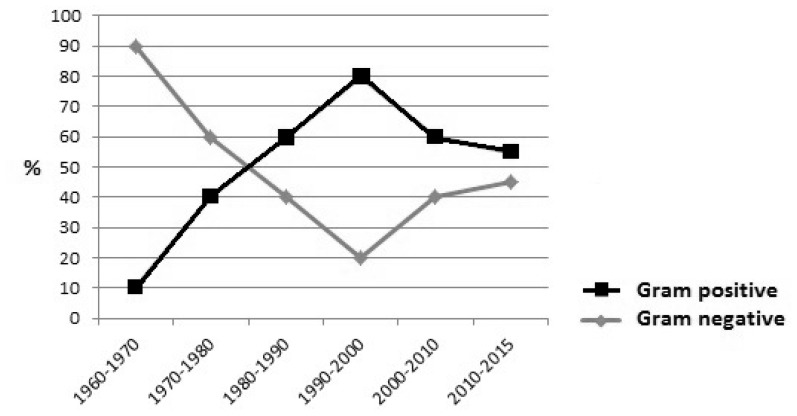
Time trend of bacterial etiology in neutropenic patients BSI.

These infections, mostly caused by bacteria, range from 11 to 38% mortality in neutropenic patients,^3^,^4^ with an unknown origin in most cases (oropharyngeal and gastrointestinal tract are assumed as probable sources). As shown in figure 1, the etiology of BSI has changed through the years. Since 1960, the importance of Gram-negative bacilli in BSI began to be clearly recognized and in the following two decades these organisms represented the most frequent etiological agents. During the nineties, Gram-positive bacteria and emerged as a leading cause of BSI. This increased prevalence has been analyzed by several authors,^5^–^7^ factors such as the large use of central venous catheters (CVC), fluoroquinolones (FQ) and antifungal prophylaxis, gut decolonization strategies, use of high cytarabine doses, use of protonic pump inhibitors have been highlighted as possible causative factors. In the last few years, many papers report a turnaround in BSI etiology, with an increasing role of gram negative bacteria,^2^,^5^,^8^ becoming the first cause of BSI in some settings.^7^

Moreover, the widespread of antimicrobial resistance, especially among Gram-negative bacilli as extended spectrum beta-lactamase (ESBL) producing Enterobacteriaceae or carbapenem resistant Gram- negative bacteria (*Acinetobacter baumannii, Klebsiella pneumoniae, Pseudomonas aeruginosa*), makes the correct setting of empirical therapy becoming a challenge, since alternative regimes are very few and often present some management issues.^1^

The aim of this paper is to review the current BSI epidemiology among neutropenic onco-hematologic patients, as well as to highlight the most important clinical features and therapeutic management issues.

## Gram-positive BSI in Hematologic Cancer Patients

Gram-positive bacteria BSIs in neutropenic patients became a major concern during the nineties because of their growing prevalence. The emergence of staphylococcal infections in relation to the increased use of CVC and FQ prophylaxis led to a significant reduction in the proportion of Gram-negative bacteria.^5^ A large prospective multicenter study by Cordonnier *et al* 6 established the Gram-positive risk index based on four major factors represented by the use of high cytarabine doses, proton pump inhibitors, decolonization strategies with colimycin without aminoglycosides and the presence of chills at the onset of fever.

Other authors also outlined the importance of high-grade mucositis and toxic enterocolitis in the development of streptococcal and enterococcal bacteremia during neutropenia.^9^–^10^ Nowadays Gram-positive bacteria still reaches 50% of BSI in neutropenic patients,^8^–^7^ being coagulase negative staphylococci (CoNS) the most frequent, followed by streptococci, *S. aureus*, enterococci, and occasionally *Corynebacterium spp* or other rare Gram-positive bacteria.

## Coagulase Negative Staphylococci (CoNS)

CoNS normally colonize mammalian skin and mucosa. In the past, they were almost universally considered as blood cultures contaminants. *S. epidermidis* has been recognized as the single most frequently isolated species from BSI. *S. haemoliticus, S. lugdunensis, S. saprophiticus, S. capitis, S. auricularis* have been isolated less frequently. In general they have a low grade virulence with a poor propensity to invade; however they have a peculiar ability to form a biofilm on biomaterials^11^ and often carry resistance genes.

CoNS are a major cause of BSI in neutropenic patients reaching 25% (5–60%) of all cases.^8^ As previously outlined, their incidence in this population seem to be related to the use of FQ prophylaxis. Gudiol *et al.* observed a significant reduction of Gram-positive BSI since FQ prophylaxis was abandoned in their center. A significant part of CoNS’s bacteremias seems to be related to mucosal more than commensal skin bacteria.^12^–^13^ This could explain their important role in neutropenic patients in which mucosal disruption is very frequent due to the cytotoxic treatment.

Even if they are the first BSI etiologic agent in neutropenic patients, their clinical relevance is questionable. Their attributable mortality is low,^14^ as for immune-competent patients in the absence of specific risk factors (such as prosthetic heart valves, joints, and other prosthetic materials).

CoNS blood isolates are usually methicillin resistant, achieving an 80% rate in the last reports^15^ except *S. lugdunensis* or *S. capitis* that are almost always susceptible to oxacillin.^16^

Concerning glycopeptides, growing resistance to teicoplanin has been observed,^15^ in particular in *S. haemoliticus* where it can reach 20% of clinical isolates.^14^ On the other hand, resistance to vancomycin is still very low, except for *S. schlefferi.*^16^ More recently an alarming emergence of linezolid resistant *S. epidermidis* has been described in Greece.^17^ Resistance to linezolid has been associated with higher virulence and higher attributable mortality compared to linezolid susceptible staphylococci^18^ but they have not been described yet among neutropenic patients. Resistance to daptomycin is still anecdotic.^19^

## Staphylococcus aureus

*S. aureus* is a common cause of both hospital and community acquired BSIs^11^ and it handles 6% (0–20%) of BSIs in onco-hematologic patients.^8^ The clinical management of *S. aureus* BSI (SAB) changes in case of complicated or uncomplicated presentation,^20^ in terms of duration of treatment, indication to perform an echocardiogram and metastatic foci research.

Surprisingly, compared to non neutropenic patients, *S. aureus* BSI during neutropenia seems to be associated with lower attributable mortality and low incidence of metastatic events or endocarditis (Table 1).^21^ Two explanations have been proposed for this phenomenon. Firstly, in neutropenia even few cells of *S. aureus* could be able to gain access to bloodstream trough altered mucosal and skin barrier and evade phagocytosis; thus an altered bacterial clearance could be responsible for positive blood cultures even with very low inoculum bacteremia. On the other hand, the absence of severe sepsis and septic shock could be related to the inability of these patients to produce the highly orchestrated inflammatory response (that include neutrophils and macrophages).^21^

Methicillin resistance among *S. aureus* isolates reported in Europe in 2013 was 18% with percentages ranging from 0 to 64% depending on the country.^22^ Neutropenic patients are at high risk to become MRSA carriers. In fact the use of FQ, recommended as prophylaxis in all cases of prolonged neutropenia,^1^ can represent an important risk factor for the emergence of MRSA.^23^

Vancomycin resistance is a marginal problem but high vancomycin MIC (between 1 and 2 mg/L), is associated with risk of failure.^24^ Interestingly vancomycin MIC >1 mg/L seem to be independently associated with the worst outcome also in methicillin susceptible *S. aureus* (MSSA) infected patients.^25^

Linezolid resistant *S. aureus* are still rarely isolated, but several reports in the last few years^26^,^27^ highlight this emerging problem that is not yet described in neutropenic population. Daptomycin resistance is also very rare and described mainly in case reports.

Because of low attributable morbidity and mortality of methicillin resistant strains, empirical treatment with glycopeptides is not required in neutropenic patients as demonstrated in two recent meta-analysis, where it was outlined that a appropriate delayed treatment had no impact on prognosis.^28^–^29^

Considering the high rate of gastrointestinal origin of staphylococcal BSI and the management problems in onco-hematologic population (piastrinopenia, chemotherapies needing a central line) the indication for the removal of CVC has to be considered for each single case. However the ascertained *S. aureus* etiology of a catheter related BSI is an absolute indication for the removal of the catheter.^30^

The antibiogram guided therapy for MRSA has to take into account that a vancomycin MIC >1 mg/L could lead to a failure when treated with vancomycin. Daptomycin should be preferred in these cases unless in the presence of pneumonia. The possible use of clindamycin, cotrimoxazole and aminoglicosides needs to be evaluated in each case, due to the variable susceptibility of these antibiotics in MRSA.

The use of newer drugs need further evaluations but should be considered for cases difficult to treat. In Table 2 are reported the newest anti-staphylococcal drugs that are already or will be soon available.

## Enterococci

Enterococci reach only 5% (0–38%) of BSIs (E-BSI) in neutropenic patients, while the higher rates are observed in hematopoietic stem cell transplantation (HSCT) recipients, in particular in the first 10 days after transplantation.^8^ Mikulska and collaborators^10^ identified risk factors associated with E-BSI in this category of patients: donors other than HLA identical, pharyngeal enterococcal colonization, high grade mucositis, Karnofsky score <50, previous use of third generation cephalosporines.

*E. faecalis* and *E. faecium* are the most frequent isolated species, but *E. faecalis/E. faecium* ratio of isolation has changed during the last 20 years. It was approximately 10:1 in the eighties^31^ and it is almost 20:1 in more recent reports;^10^,^32^ this is probably due to *E. faecium* resistance profile.

The clinical significance of their isolation (poor clinical condition marker versus “true infection maker”) should be established in each cause of BSI during neutropenia.

Enterococci intrinsic virulence is low but they are intrinsically resistant to aminoglicosides, cotrimoxazole (in vivo), cephalosporines.^11^ Ampicillin resistance is very prevalent for *E. faecium* and rare for *E. faecalis*. High level aminoglycosides resistance is very prevalent for both *E. faecalis* and *E. faecium*. Resistance to vancomycin is mainly present in *E. faecium*. The most frequent genes being Van A and Van B that codify for modified cell wall proteins. In Van B strains teicoplanin is active but not in Van A strains. Vancomycin resistance is more frequent in Eastern Europe, UK and USA.^22^ Factors associated with vancomycin resistant enterococci BSI (VRE-BSI) among E-BSI are the recent use of vancomycin or glucocorticosteroids or severity of illness.^33^ In HSCT recipients, previous VRE colonization and Graft versus Host Disease (GVHD) were also associated with VRE-BSI.^34^

VRE seem to a have a peculiar clinical behavior compared to VSE (Vancomycin Susceptible Enterococci). Diaz Granados and collaborators performed a meta-analysis including 1614 E-BSI cases, and highlighted an increased mortality in VRE-BSI, compared to VSE-BSI (OR 2.51).^35^ The authors could not conclude if this observation was an effect of a delay of appropriate therapy or of an increased virulence of VRE (that are *E. faecium* in most cases).

Empirical treatment for E-BSI is not recommended. No benefit was observed even in HSCT recipients colonized with VRE receiving empirical linezolid.^36^

Even if randomized controlled trials comparing linezolid and daptomycin in VRE-BSI are lacking, the available evidence suggests a superiority of linezolid in terms of mortality and treatment failure.^37^–^38^ This observation has also been highlighted in neutropenic patients.^39^

## Viridans Streptococci

Viridans streptococci are an important part of the normal microbial flora. They are indigenous to the upper respiratory tract, the female genital tract, and all regions of the gastrointestinal tract but are most prevalent in the oral cavity.^11^ They normally have a low virulence and tendency to invade. However not surprisingly they are an important cause (5%) of BSI in neutropenic patients.^8^

In the previously mentioned paper, Cordonnier established a score of risk for the development of viridians streptococcal BSI in neutropenic patients. This score included the use of high dose cytarabine during induction therapy, oral colimycin without aminoglycosides as decontamination, prophylaxis with antifungal drugs and the presence of diarrhea.^6^ Oral mucositis appeared associated with this infection only in univariate analysis. Another possible association has been seen with periodontitis at the time of the onset of neutropenia.^40^

Viridans streptococci BSIs (VS-BSI) during neutropenia carry substantial morbidity and mortality. Attributable mortality is ranging from 6 to 12%.^11^ Severe cases, presenting ARDS or shock or both were associated with allogenic bone marrow transplantation, presence of severe oral mucositis (grade 3 or 4) and high dose therapy with cyclophosphamide reaching 11% of the streptococcal bacteremias in Marron *et al* series.^41^ Since the end of the eighties, reduced susceptibility (MIC > 0.12mg/L) and resistance to penicillin (> 0.25 mg/L) have been described in viridians streptococci,^41^–^43^ including those isolated in onco-hematologic patients.^11^ Poor susceptibility of streptococci to ceftazidime^41^ should suggest not using this agent as an empirical treatment of febrile neutropenia in institutions, and should preclude its use in patients at high risk of streptococcal BSI.^41^

## *Corynebacterium spp.* and other Rare Gram-Positive Etiologies

**Figure 2:**
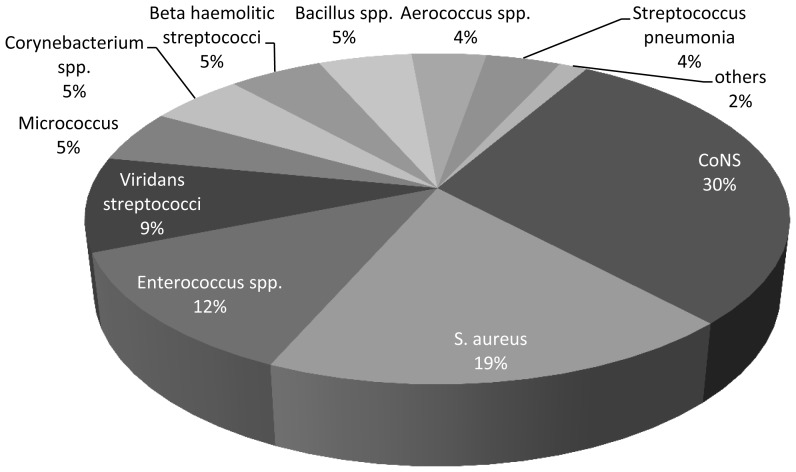
Spectrum of gram + bacteremias in patients with cancer (1082 patients) *Modified by Rolston*^44^

“Other Gram-positive” etiologies reach 6% (0–21%) of BSI in neutropenic patients.^8^ They include Corynebacteria (usually represented by multidrug resistant (MDR) isolates with a spectrum of antibiotic resistances similar to that of MRSA,^9^ beta haemolytic streptococci and several organisms that colonize the skin such as *Aerococcus spp*., *Bacillus spp*., *Micrococcus spp*. and *S. pneumonia* are also relevant. Organisms, such as *Listeria monocytogenes, Rhodococcus equi,* and vancomycin-resistant bacteria, such as *Lactobacillus spp*., *Leuconostoc spp*. and *Pediococcus,* are occasionally encountered (Figure 2).^44^,^45^ Both linezolid and daptomycin demonstrated good in vitro activity against all Gram-positive isolates in cancer patients.^44^

## Gram-negative BSI in Hematologic Cancer Patients

We already mentioned in the introduction the turnaround in BSI etiology in neutropenic patients. In a recent Italian multicenter study,^7^ Gram-negative bacteria were the most frequent isolates in patients with hematologic malignancy. Infections caused by these microorganisms have been identified as an independent predictor of death in patients with malignancies and bloodstream infection: BSI alone reach 12%–42% of mortality.^46^,^47^

The distribution of Gram-negative bacilli from BSI remained stable over time but the emergence of multi drug resistant (non susceptible to more than 1 agent in 3 or more antimicrobial categories, MDR), extremely drug resistant (non susceptible to more than 1 agent in all but 2 or less antimicrobial categories, XDR) and pandrug resistant (non susceptible to all antimicrobial active agents, PDR) isolates represent today the main challenge in managing of Gram-negative BSI.^48^

## Escherichia coli

Due to its ability to colonize the human gastrointestinal tract, *E. coli* is the most common bacterial species found in human fecal flora. Thus, it is not surprising that it also represents the more frequent cause of Gram-negative BSI in neutropenic patients, reaching almost one quarter of all isolates.^8^,^44^,^49^,^50^ In this patient population, the morbidity and mortality due to *E. coli* BSI might be due to several factors, including antibiotic resistance, FQ resistance and ESBL production, which are the most represented, can be present in almost one third of all isolates and both are favoured by the widespread use of FQ prophylaxis.^5^,^51^–^54^

Since inadequate initial antimicrobial therapy has been associated with poorer outcomes, in recent years carbapenems have been employed increasingly as agents of choice against ESBL *E. coli* and for the empirical treatment of BSI in neutropenic patients.^50^,^55^ However, the concomitant emergence of carbapenems resistance GNB and the observation that carbapenem restriction might be associated with lower rates of carbapenem-resistances have led researchers to consider carbapenem-spearing antibiotic strategies.^56^

Among the potential alternative therapies explored, the role of piperacillin-tazobactam, has been reassessed: in fact patients treated with this combination and those treated with carbapenem against β-lactam-β-lactamase inhibitor BLBLI susceptible *E. coli* presented a similar therapeutic outcome.^51^,^57^

Therefore, considered these assumptions, in a setting of high ESBL prevalence, a possible antimicrobial stewardship program could be based on simply model of de-escalation strategy. Indeed, in case of proven susceptibility, the change of treatment from carbapenem to piperacillin/tazobactam could be safe and prevent the risk of carbapenemase induction

Otherwise in epidemiologic settings characterized by a high prevalence of infection due to piperacillin-tazobactam resistant *E. coli*, empirical therapy with a combination piperacillin-tazobactam and tigecyclin could be another suitable option.^58^

## Klebsiella pneumoniae

*K. pneumoniae* is the primary species of genus *Klebsiella* associated with illness in human beings. It is found in the gastrointestinal tract and is frequently involved in health-care and intensive care unit (ICU) associated infections.^59^ Infections with *K. pneumoniae* are usually hospital-acquired, sustained by MDR strains and occur primarily in patients with impaired host defenses.

As described for *E. coli*, *K. pneumoniae* is often represented by FQ and third generation cephalosporin resistant strains; thus it shares with *E. coli* all the therapeutic challenges deriving from these types of resistance. Moreover, several mechanisms have been identified as responsible for carbapenem resistance among Enterobacteriaceae: Ambler class A beta-lactamases are enzymes that can be either plasmid encoded (*bla*_KPC_ and, less frequent, *bla*_IMI-2_, *bla*_GES_) or chromosomally encoded (*bla*_NMC_, *bla*_SME_, *bla*_IMI-1_, *bla*_SFC-1_). The class B metallo-β-lactamases includes (MBLs) Verona integron-encoded metallo-β-lactamase (*bla*_VIM_), *bla*_IMP_, and the New Delhi metallo-β-lactamase (*bla*_NDM_). *Bla*_OXA-48_ carbapenemases belong to Ambler class D. Finally resistance to carbapenems can also be caused by hyperexpression of *AmpC* gene or to decreased permeability of the outer membrane because of porin loss in combination with the expression of AmpC enzymes or ESBLs.^60^,^61^

From an epidemiological point of view, *K. pneumoniae* represents the third leading cause of GNB BSI in neutropenic patients population reaching almost 12.5% in a recent Italian multicenter study.^7^ Since 2008 an increasing number of reports described the spread of carbapenem resistant strains mainly in the Mediterranean and Southern European countries with a rapid spread in Israel and Greece.^62^ The spread of carbapenem resistant Enterobacteriaceae (CRE) has dramatically increased also in Italy rising from 15.2% in 2010 to 34.3% in 2013.^7^,^63^,^64^

The high incidence of these MDR strains in immune-compromised populations was confirmed by recent multicenter studies reporting that a KPC producing *K. pneumonia* (KPC-Kp) rectal colonization was common in onco-hematological patients. In this cohort the colonization was followed by an infection in 39.2% cases of allogeneic Stem Cell Transplantation Recipients (allo-SCT)^65^ and 45% of cases of neutropenic patients.^66^ Observed mortality rate attributable to KPC-Kp BSI was of 57.6%. in adult inpatients,^64^ while it was of 64.4% in allo-STC recipients.^65^ Therefore considering these aspects, it is crucial to recognize the KPC-Kp carriers and consider this information in febrile neutropenic patients at risk of BSI.

**Figure 3:**
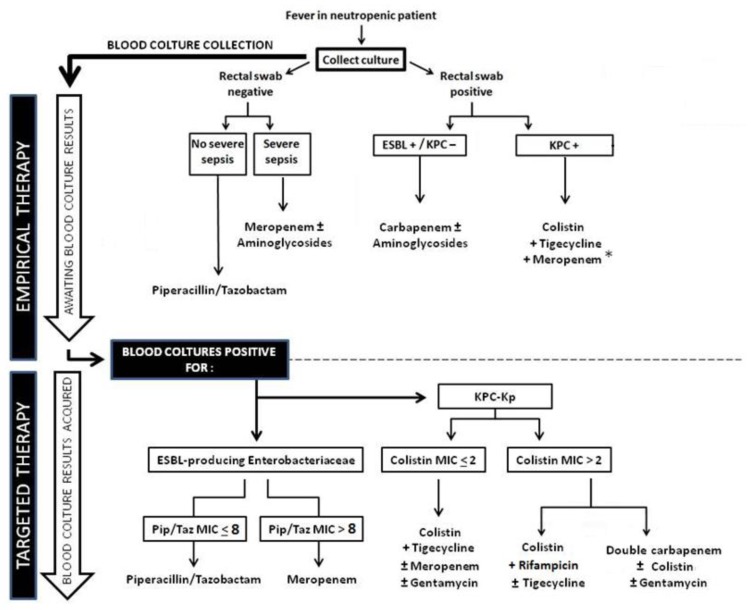
Flow chart for empirical and targeted treatment of febrile neutropenic patients at risk of ESBL and or KPC producing Enterobacteriace (Colistin: 9 M loading dose, 4,5 M q 12h; Rifampicin: 600 mg q 24h; Gentamicin: 5–7 mg/kg; Doripenem: 500 mg q 8h, extended infusion; Meropenem: 1–2 gr q 6–8h, extended infusion; Ertapenem 1 gr q 24h; Tigecycline: 200 mg loading dose, 100 mg q 12h; *in clinical center with blood isolate meropenem MIC≥16 consider gentamycin instead of carbapenem).^96^–^106^

Although empirical treatments against KPC-Kp are not recommended by current IDSA-ECIL guidelines, due to their potential toxicity and off label usage (Figure 3), we believe that these therapies are justified in this setting, according to the evidence of the literature. The different scenarios potentially met by the clinician are analyzed in the figure 3, which provides a diagnostic and therapeutic algorithm that might be useful in this setting. In any case, it is essential to stress that neutropenic patients should be routinely screened on rectal swab cultures to identify patients with KPC-Kp gut colonization.

Awaiting new drugs showed in Table 2 potentially active against KPC-Kp, at the moment colistin represents the back-bone of therapeutic regimes against KPC-KP;^64^,^67^–^70^ its use is possible in the case of infections due to both colistin susceptible and resistant strains, as showed in figure 3.

Synergistic activity have been reported with therapeutic strategies combining colistin with rifampicin^71^ and ertapenem with meropenem +/− colistin.^72^ This last strategy, so called “double carbapenem” therapy, could be employed in cases of severe infection not responsive to previous treatment. The activity of combination is justified from *in vivo* studies^73^–^74^ that seems to corroborate *in vitro* experiments performed by Bulik et al., who recently postulated that the enhanced efficacy of this therapy against KPC-Kp may be related to the KPC enzyme’s preferential affinity for ertapenem.^75^

Finally, it is worth to remember that rectal swab surveillance is recommended as a component of infection prevention programs and of antimicrobial stewardship that can reduce the rate of CRE infections, including BSI.

## *Pseudomonas aeruginosa* and other non Fermentative Gram Negative Bacilli (NFGNB)

*P. aeruginosa* is an ubiquitary Gram-negative invasive pathogen, responsible for severe infections in immune-compromised hosts. Since 1960 *P. aeruginosa* BSI has been highlighted as an important and frequent cause of morbidity and mortality in neutropenic patients. With the introduction of FQ prophylaxis *P. aeruginosa* prevalence progressively declined, but nevertheless it is still responsible for 18% to 27% of BSI in this population^2^,^5^,^7^ with a mortality rate of 40%.^76^

However, at the moment the benefit of FQ prophylaxis, despite its historical value, is a matter of concern for its association with the emergence of antibiotic resistance, especially in those countries where MDR and XDR strains reached 50% of isolates.^63^ Furthermore the problem of the emergence of MDR strains is related to the clinical outcome. In fact, an increased invasive capacity of MDR *P. aeruginosa* was evidenced by the observation that the patients colonized with MDR strains are at higher risk of BSI compared to those with a no-MDR colonization.^77^

Concerning therapeutic resources, first line recommended therapy of ECIL and IDSA guidelines for the management of fever in neutropenic patients ensure coverage for susceptible *P. aeruginosa*. The addition of aminoglycosides could be effective in cases of severe sepsis or septic shock*.*

Regimes based on colistin, in association with rifampicin +/− antipseudomonal carbapenem, have been suggested to treat MDR/XDR strains due to their possible synergistic effect.^78^,^79^ Among soon available drugs, ceftolozane-tazobactam, seems to be the most promising in the treatment of such infections.^80^

NFGNB only account for less than 3% of BSI in neutropenic patients. In this group, *Stenotrophomonas maltophilia* and *Acinetobacter baumannii* are the most represented bacteria (see Table 3).

Ecthyma gangrenosum (EG) is a well-recognized cutaneous infection classically associated with *S. maltophilia* and *P. aeruginosa* bacteremia. EG usually occurs in patients who are critically ill and immunocompromised; it is almost always a sign of pseudomonal or stenotrophomonal sepsis.^81^,^82^ Intrinsic resistance profile of *S. maltophilia* is a therapeutic hitch worthy of consideration: trimethroprim-sulfametoxazole is the drug of choice, but also levofloxacine and moxifloxacine were usually active.^83^,^84^ Moreover recently i.v. minocycline demonstrated an exellent in vitro activity.^85^

*Acinetobacter spp* BSI accounts for only 1% of neutropenic patients. Therapies versus *A. baumannii* are based on carbapenem or aminoglycosides when the strains are susceptible to these drugs and colistin, eventually in association with rifampicin and ortigecyclin, ampicillin-sulbactam or carbapenem, when the stains are XDR.^86^–^91^

In conclusion, the current epidemiology of BSI in onco-hematologic patients is characterized by the emergence of MDR pathogens. This observation has several implications both in the institution of empirical and targeted treatment and in the need of containment strategies. We proposed here some possible regimens for empirical and focused treatment based on current evidence to help the clinician who is going to treat febrile neutropenia in the MDR bugs era. We believe that every effort has to be made for the containment of the spread of this pathogens. For this purpose, shared antibiotic stewardship strategies need to be implemented. Concepts like antibiotic de-escalation, availability of the antibiograms, isolation of the colonized patients, and careful limitation of carbapenem use are cornerstones of resistance containment both in neutropenic and non-neutropenic patients.

Some special considerations should be made on neutropenic patients. First of all, FQ prophylaxis has been highlighted as one of the most important causative factors for the emergence of ESBL enterobateriaceae and MRSA. Therefore, its use need probably to be systematically re-evaluated at least in selected epidemiological settings (i.e. in relation to FQ resistance prevalence among *E. coli* isolates). Secondly, around 70% of fevers in neutropenia are classified as fever of unknown origin (FUO)^92^ in which antibiotic therapy could be unneeded. Expert opinion^93^ and recent evidence^94^ support early discontinuation of antibiotic therapy in FUOs. Finally, approaches to reduce the antibiotic exposure with the adoption of short antibiotic treatments for specific infections should be evaluated. As an example, a five days course of daptomycin for CoNS BSIs promptly responding to CVC removal might prove efficacious.

The knowledge of the general and local epidemiology and resistance profiles are of a paramount importance in the correct management of febrile neutropenia. Frequent, up to dated, reports about trends in etiology and emerging resistances need to be implemented.

## Figures and Tables

**Table 1:** Severity of SAB in neutropenic and non neutropenic patients.

Variable	Neutropenic (36)	Non neutropenic (36)	p-value
Severe sepsis or septic shock	1	10	0,002
Duration of bacteremia	1,33	1,95	0,03
Metastatic foci	0	5	0,002
Attributable mortality	1	9	0,006

**Table 2:** New or soon available drugs for MDR bacteria and their characteristics

Agent (class)	Spectrum	Route of administration and dosage
Quinupristin-dalfoprisitin (streptogramin)	Streptococci, MR-Staphylococci, VRE, Corynebacteria, *L. monocytogenes, N. meningitidis, M. catarrhalis, Chlamydophilaspp, M. penumoniae*	Intravenous, central line only. 7,5 mg/kg q8–12h
Telavancin (lipoglycopeptide)	Streptococci, MR Staphylococci, VSE, Corynebacteria*, L. monocytogenes,* Clostridia, *Actinomyces, Peptostreptococcus*	Intravenous, 10 mg/kg q 24h. Infusion over 1h
Dalbavancin (lipoglycopeptide)	Streptococci, MR Staphylococci, VSE	Intravenous, 1000 mg once (followed by 500 mg every week). Infusion over 30 min
Tedizolid phosphate (oxazolidinone)	Streptococci, MR Staphylococci, VRE	Intravenous, 200 mg q 24h
Ceftaroline (V generation cephalosprine)	MR Staphylococci, gram negative bacteria	Intravenous, 600 mg q 8–12h over 1 h
Ceftazidime – avibactam	Broad spectrum anti BGN, including ESBL, KPC and enhanced activity against *P. aeruginosa*. Limited activity vs anaerobes	Intravenous, 2/0,5 gr q 8h 2 h infusion
Ceftozolozane/tazobactam	Broad-spectrum anti BGN, ESBL, *P. aeruginosa.* No activity vs KPC & MBL. Limited activity vs anaerobes	Intravenous, 1,5–3 gr q 8h
Imipenem MK 7655	Broad spectrum anti BGN, including ESBL, KPC and enhanced activity against *P. aeruginosa*. Optimal activity vs anaerobes	Intravenous, 0,5/0,25 (or 0,125) q 6h
Meropenem RPX 7009	Broad spectrum anti BGN, including ESBL, KPC. Optimal activity vs anaerobes	Intravenous, 2/2 gr q 8
Plazomicin	Broad spectrum vs gram positive (MRSA, VISA) and gram negative *P. aeruginosa*, ESBL, carbapenemase (MBL)	Intravenous

**Table 3:** Spectrum of gram negative infection in neutropenic patients and principal type of resistance.

Organism	Frequency	Type of resistance
*E. coli*	18–45%	ESBL, FQR
*Klebsiella spp*	11–18%	ESBL, FQR, KPC, MDR
Other Enterobacteriaceae	15–18%	ESBL, FQR, KPC, MDR
Pseudomonas aeruginosa	18–24%	FQR, MDR
*Stenotrophomonas maltophilia*	2,5%	MDR
*Acinetobacter spp*	<3%	MDR
